# Climate change alters the structure of arctic marine food webs due to poleward shifts of boreal generalists

**DOI:** 10.1098/rspb.2015.1546

**Published:** 2015-09-07

**Authors:** Susanne Kortsch, Raul Primicerio, Maria Fossheim, Andrey V. Dolgov, Michaela Aschan

**Affiliations:** 1 Norwegian College of Fishery Science, UIT the Arctic University of Norway, 9037 Tromsø, Norway; 2 Institute of Marine Research (IMR), 9294 Tromsø, Norway; 3 Knipovich Polar Research Institute of Marine Fisheries and Oceanography (PINRO), 6 Knipovich Street, 183038 Murmansk, Russia

**Keywords:** biogeography, climate warming, fish community, modularity, network structure, food web topology

## Abstract

Climate-driven poleward shifts, leading to changes in species composition and relative abundances, have been recently documented in the Arctic. Among the fastest moving species are boreal generalist fish which are expected to affect arctic marine food web structure and ecosystem functioning substantially. Here, we address structural changes at the food web level induced by poleward shifts via topological network analysis of highly resolved boreal and arctic food webs of the Barents Sea. We detected considerable differences in structural properties and link configuration between the boreal and the arctic food webs, the latter being more modular and less connected. We found that a main characteristic of the boreal fish moving poleward into the arctic region of the Barents Sea is high generalism, a property that increases connectance and reduces modularity in the arctic marine food web. Our results reveal that habitats form natural boundaries for food web modules, and that generalists play an important functional role in coupling pelagic and benthic modules. We posit that these habitat couplers have the potential to promote the transfer of energy and matter between habitats, but also the spread of pertubations, thereby changing arctic marine food web structure considerably with implications for ecosystem dynamics and functioning.

## 1. Introduction

Arctic marine ecosystems are exposed to rapid environmental change driven by accelerated warming [1,2]. Changes in habitat characteristics, such as reduced sea ice coverage and increased seawater temperature induce substantial food web reorganizations via regional gains and losses of species, potentially triggering cascading effects [3,4]. Temperature influences the distributions of marine organisms and recent poleward movements of boreal species have been documented as a response to warmer ocean temperatures and reduced sea ice coverage [5–7]. As a consequence, novel interactions will be established between incoming and resident species with implications for food web configuration and ecosystem functioning [8].

Food webs are structurally complex and contain a variety of sub-modules that may be treated as functional units [9–11]. Modularity refers to sub-groups of prey and predators interacting to a greater degree with each other than with species from other sub-groups, and is a food web property with implications for the dynamics and functioning of ecosystems [10]. According to network theory, food webs with greater modularity should be more persistent, as any pertubation effects may be retained within the modules, delaying or stopping their propagation to other modules [10,11]. Individual species can play different roles with respect to modularity, depending on how many feeding links they have within their own module and/or across modules [12].

From a network perspective, the ecological role of a species is a direct result of its position in the food web, the number of interactions it has with neighbouring species and their interactions, and also the strength of these interactions [13,14]. Some species are functionally more important than others and may have disproportionately large effects on food web structure [15]. Central and functionally unique species include keystone species, key species, ecosystem engineers and network hubs [16–18]. Theoretical and empirical results suggest that network hubs, or super-generalists, connect modules and communities due to their wide niche breadth, environmental tolerance, apex position in local communities and high motility [19–21]. If species affected by pertubations possess key functional roles in the food web, then the potential higher order, indirect effects of those pertubations on the entire food web structure can be dramatic.

A species' response to climate warming depends on several classes of traits, affecting its sensitivity and adaptability [22]. Among these traits, body size and feeding behaviour (e.g. generalist versus specialist) have particular relevance to food web structure. Large, migratory generalists are expected to respond rapidly to climate warming as they can easily move into new suitable regions, where they can exploit available niches and prey. Recent evidence shows that highly migratory, generalist fish are indeed moving poleward and entering arctic marine food webs [5,23–26]. To understand the implications of these poleward movements for ecosystem functioning and vulnerability, it is crucial to investigate how boreal taxa influence the structure of arctic marine food webs.

Network research offers a framework and the tools for characterizing the structure of food webs and the functional importance of species in ecosystems undergoing change [13]. Here, we use a network approach to explore and compare general structural properties of highly resolved boreal and arctic food webs of the Barents Sea. On the food web (network) level, we focus on structural properties with particular importance for food web dynamics such as the degree of modularity. On the species (node) level, we focus on taxa with central functional roles in the network. Further we evaluate potential changes in arctic marine food web structure due to poleward shifts of boreal species driven by climate warming. We ask the following questions: (i) what are the structural differences between boreal and arctic food webs of the Barents Sea? (ii) What roles do different taxa play with respect to modularity? (iii) How are the ongoing poleward shifts of boreal fish affecting the structure of arctic marine food webs?

## 2. Material and methods

### (a) Study area and occurrence data

The Barents Sea is a large, open sub-arctic shelf sea bordering the Arctic Ocean (electronic supplementary material, figure S1). We defined a boreal and an arctic study region within the Barents Sea based on hydrology and species distributions (electronic supplementary material, figure S2*a,b*). We chose areas southwest and northeast of the polar front, the main hydrological demarcation separating boreal and arctic biogeographic regions of the Barents Sea (electronic supplementary material, figure S2*a*). Information on the occurrence of taxa within the defined study areas was obtained from data sampled by the joint Russian–Norwegian Ecosystem Survey in the Barents Sea. Since 2004, this survey has annually sampled plankton, fish and benthos at station level, and sea birds and marine mammals along transects in August–September when sea ice extent is at its minimum [27].

Fish and epibenthos were allocated to the study regions based on station-wise (approx. 300 fish stations, approx. 40 epibenthos) presence/absence data. Occurrence of marine mammals, sea birds and zooplankton were assigned to specific sub-regions (see polygons in the electronic supplementary material, figure S2b) based on recordings from the Ecosystem Survey, and then assigned to the relevant study regions. All basal taxa were designated present in both regions, with the exception of sea ice algae, which are only included in the arctic marine food web. Results from the joint Russian–Norwegian Ecosystem Surveys can be found in several reports and research papers [5,27–29]. See the electronic supplementary material (appendix S1) for a more detailed description of the Barents Sea region, the boreal and the arctic study regions and the sub-sampling of taxa within these regions.

### (b) The food webs

Food webs consist of trophospecies, i.e. groups of organisms (nodes) sharing the same predators and prey, and their feeding links [30]. In our food webs, individual trophospecies usually correspond to taxonomic species, but can sometimes refer to higher taxonomic groups, e.g. genus, family and class. The food web (meta-web) encompasses the most common taxa in the Barents Sea from the seafloor to the surface, comprising 233 trophospecies and 2192 feeding links. The food web includes detritus and bacteria, eight basal taxa, 43 zooplankton, 79 benthic and 77 fish trophospecies, as well as nine sea birds and 15 marine mammals. See the electronic supplementary material, tables S1 and S2, for exhaustive lists of all taxa included in the boreal and the arctic food webs, their degree (no. of trophic interactions), topological role, and habitat and functional group affiliation (e.g. basal taxa, zooplankton, benthos, fish, sea birds and marine mammals). Further details on how the food web was assembled and on the strength and limitations of the dataset are presented in the electronic supplementary material (appendix S1) and in Planque *et al.* [31]. The dataset files of the meta-web can be downloaded from the *Ecological archives* website [31].

The sub-webs specific for the boreal and the arctic regions were constructed by choosing subsets of taxa according to their occurrence (presence/absence) in the respective regions based on the Barents Sea Ecosystem Survey data (see further details in the electronic supplementary material, figure S2). The arctic marine food web is representative for the beginning of the recent warming period in the Barents Sea characterized by sea ice retraction and increasing water temperatures. The trophic interactions specific to the sub-regions were sub-sampled from the meta-web, assuming that species co-occurring in the sub-sampled regions and connected via trophic interactions in the meta-web, will also interact in the sub-webs. The two food webs differed with regard to trophospecies composition (127 unique and 106 shared trophospecies, see the electronic supplementary material, figure S3).

To evaluate the effect of the poleward movements of boreal fish on food web structure in the Arctic, we updated the arctic food web (hereafter referred to as arctic II) by including four fish species: atlantic cod (*Gadus morhua*), haddock (*Melanogrammus aeglefinus*), golden redfish (*Sebastes norvegicus*) and beaked redfish (*Sebastes mentella*). These fish were chosen because they have boreal affinities and display substantial responses to climate warming in terms of poleward shifts and biomass increases in the arctic region of the Barents Sea [5,23]. The trophic interactions between cod, haddock and the two redfish and other trophospecies in the arctic II food web were sub-sampled from the meta-web, assuming that trophospecies will interact in the sub-webs, if they interact in the meta-web. The boreal and the arctic Barents Sea food web files used in this study can be downloaded from the Dryad repository (doi:10.5061/dryad.73r6j).

### (c) Topological properties

To characterize the structure of our food webs, we estimated 12 food web metrics related to properties commonly addressed by topological food web analyses [32], including modularity (Mod). Number of species (S), number of trophic links (L), linkage density (LD, i.e. number of links per species) and connectance (C, i.e. the fraction of realized links). These are standard food web metrics that capture the fundamental complexity of food web structure. Other commonly reported metrics include: percentage of trophospecies in loops, percentage of cannibals (self-loops), mean path length, mean clustering, mean omnivory and mean trophic level. The above metrics convey information about structural properties of food webs with implications for ecosystem dynamics and functioning [13,32]. See the electronic supplementary material, table S3, for abbreviations, short definitions of the food web metrics and their references. The number of loops was calculated using the software Network3D [33].

### (d) Degree distributions

The degree of a trophospecies refers to its total number of feeding links with other species and is used as a measure of food web centrality (i.e. degree centrality). Species with many connections, i.e. high degree and central species (hubs), tend to have a large impact on overall food web structure and functioning [34]. In a directed network such as a food web, the degree of a trophospecies can be decomposed into its in-degree and out-degree. In-degree refers to the number of links directed towards a trophospecies, which is the total number of its prey (i.e. its generality). The out-degree is the number of outgoing links, which is the total number of predators of a trophospecies (i.e. its vulnerability). The cumulative out- and in-degree distributions were calculated to compare the generality and the vulnerability of the trophospecies among the food webs.

To investigate how the average degree centrality has changed in the Arctic due to the poleward movement of boreal, generalist fish, we mapped the mean degree centrality at station level for the years 2004 (491 stations) and 2012 (377 stations) based on 51 fish taxa. The year 2004 is representative of the species composition and food web structure in the arctic Barents Sea in the early stage of warming experienced during the last decade [5]. The station-wise mean degree centrality calculations of fish were based on the degrees of fish in the Barents Sea meta-web. To help visualize and compare the spatial patterns, we interpolated the mean degree on a regular grid by universal kriging [35], and colour-coded the results in the Barents Sea maps. The size of the grid was 50 × 50 km (approx. 27 nautical miles) to ensure at least one station per grid cell. See the electronic supplementary material, figure S4 for annual degree centrality maps between 2004 and 2012, and electronic supplementary material, table S4 for a list of the 51 fish taxa included in the analysis.

### (e) Modularity analysis

For each food web, we calculated modularity, describing how densely sub-groups of species interact with one another compared to species from other sub-groups [36]. To find the best partition, we used the simulated annealing algorithm proposed by Reichardt & Bornholdt [37], a stochastic optimization approach that identifies modules by maximizing a modularity function [12]. For a given partition of a food web, the index of modularity *M* is defined as:
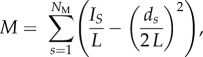
where *N*_M_ is the number of modules *s*, *I* is the number of links between nodes in module *s*, *L* is the number of links in the network and *d_s_* is the sum of degrees of all species in module *s*. The modularity value *M* lies in the interval [0, 1] and for a random partition *M* equals 0. To test whether our empirical networks were significantly more modular than random networks, we compared the modularity of our empirical networks with the modularity of 1000 randomized networks constrained by the same species degree distribution as the empirical network. To address whether the modules were associated with habitat use (pelagic or benthic) and short-weighted trophic level of component trophospecies [38], we applied linear discriminant analyses (LDA) [21]. The habitat affiliation of each trophospecies was coded B (benthic), P (pelagic) and BP (benthopelagic). Each LDA was followed by a permutation test to assess the significance of the association.

### (f) Topological roles of the species

We estimated the topological role of each species based on their module membership. We relied on module membership identified by a randomly chosen replicate of the simulated annealing algorithm. The role is described by two parameters: (i) the standardized within-module degree *z* and (ii) the among-module connectivity participation coefficient PC. The *z*-score reflects how well a species is connected to other species inside the module relative to other species within its own module. The PC parameter estimates the distribution of a species' connections across the modules. The *z*-score is defined as
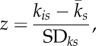
where *k_is_* is the number of links from species *i* to other species in its own module *s* and 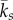 and SD*_ks_* are the average and standard deviation of *k_is_* over all species in *s*. The role of a species can also be described by its links to species in modules other than its own. The among-module connectivity PC can be defined as the number of links from species *i* to species in other modules, normalized by the degree (*k_i_*) of species *i*:
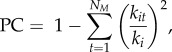
where *k_i_* is the number of links to or from species *i* and *k_it_* is the number of links from species *i* to species in module *t*. Owing to the stochastic nature of the module detection algorithm, we estimated and plotted the mean and 95% CI of the 1000 within-module (*z*) and 1000 among-module (PC) role affiliations.

The *z-*PC parameter space is divided into four regions modified from Guimera *et al.* [12]. The thresholds that define the topological roles are *z* = 2.5 and PC = 0.625. If a species has at least 60% of its links within its own module then PC < 0.625. If a species has *z* ≥ 2.5 and PC < 0.625, it is classified as a module hub, having many links within its own module. Species that are in the region *z* < 2.5 and PC < 0.625 are called peripherals or specialists. These are species that have relatively few links and most of their links are within their own module. Species that are in the region *z* < 2.5 and PC ≥ 0.625 are module connectors. Species in the region *z* ≥ 2.5 and PC ≥ 0.625 are hub network connectors. These species are characterized by high within- and between-module connectivity and are classified super-generalists.

All data analyses were performed in the statistical software R v. 3.1.0. The degree distributions and modularity were calculated using the R package ‘igraph’. The kriging of the degree centrality of fish was performed using the R package ‘gstat’, and the permutation tests associated with the LDAs were calculated using the R package ‘vegan’.

## 3. Results

### (a) Topological properties

**Figure 1.**
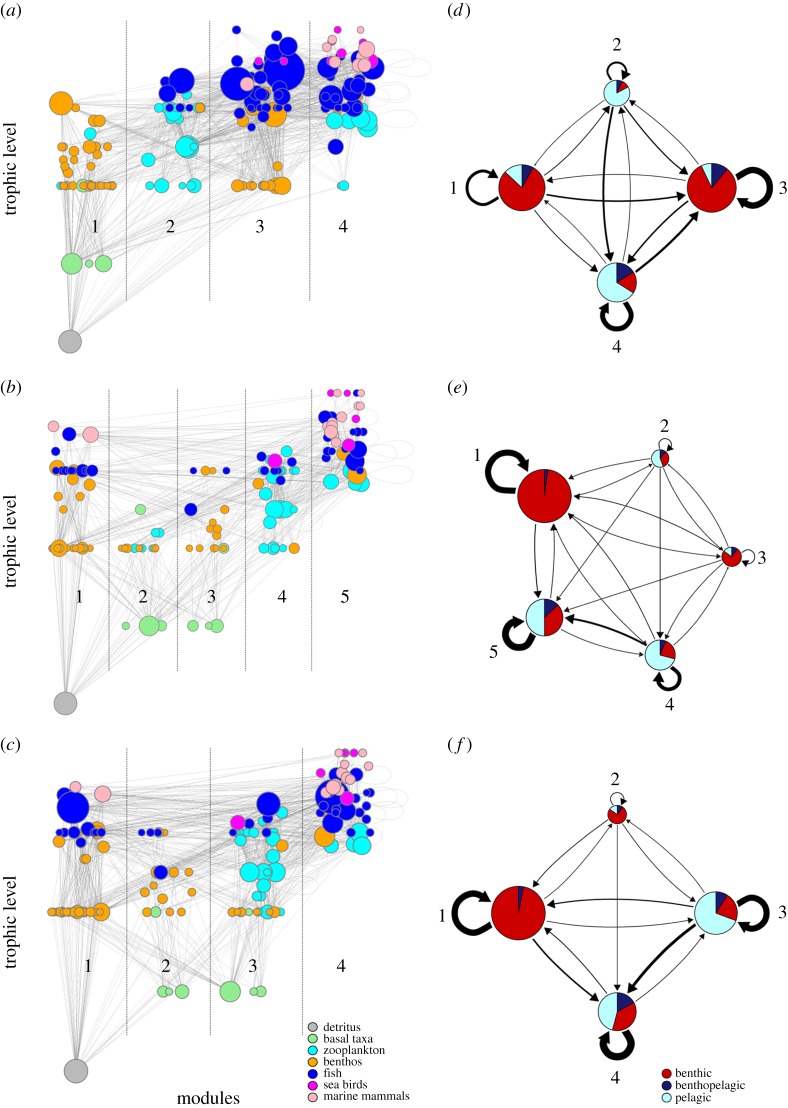
Food web diagrams of the Barents Sea for the (*a*) boreal, (*b*) arctic and (*c*) arctic II food webs. Each dot (node) represents a trophospecies. The lines connecting the nodes represent the feeding links between the trophospecies. The vertical position of the nodes indicates the trophic position of a species, and the horizontal position indicates the module affiliation of a species. The size of the nodes are proportional to the degree (no. of feeding links) of a species. The colour of the nodes indicates which functional group a trophospecies belongs to: grey, detritus; green, basal taxa; cyan, zooplankton; orange, benthos; blue, fish; magenta, sea birds; light pink, marine mammals. Schematic food web diagrams of the modular structure of the Barents Sea food webs: (*d*) boreal, (*e*) arctic and (*f*) arctic II food web. Each node (circle) represents a module in the corresponding food web. The size of the nodes indicates the number of trophospecies within each module. The colour of the nodes (pie charts) indicates the habitat affiliation of the trophospecies within the module: light blue, pelagic; red, benthic; dark blue, benthopelagic. The arrow width is proportional to the number of feeding links between modules in the direction of the arrowhead.

The Barents Sea food webs differed with regard to structural properties and modularity (figure 1). Number of trophospecies was somewhat higher in the boreal (180) compared to the arctic marine food web (159), partly due to the higher fish species richness in this region (electronic supplementary material, figure S3*a*). The boreal food web with its 1546 links had nearly double as many feeding links as the arctic food web with its 848 links (table 1). Complexity measures such as links per species and connectance were, respectively, 38% and 40% higher in the boreal food web (table 1). The boreal food web also had higher clustering (32%) and more cannibals (54%), and contained many loops (13%), whereas the arctic food web contained no loops. We re-analysed the arctic food web after including four boreal poleward moving fish (cod, haddock, golden redfish and beaked redfish) to evaluate their effect on the arctic food web structure. The inclusion of these fish species resulted in the structural descriptors of the arctic food web becoming more similar to the boreal food web (table 1). Modularity decreased by 17%, whereas connectance, linkage density, clustering and number of cannibals increased by 25%, 19%, 19% and 25%, respectively. The arctic II marine food web also contained 3% species in loops.

**Table 1.** Topological properties of boreal and arctic food webs of the Barents Sea. The arctic II food web contains four poleward moving fish: cod (*Gadus morhua*), haddock (*Melanogrammus aeglefinus*) and two redfish species (*Sebastes norvegicus* and *Sebastes mentella*). The last two columns show the percentage difference for each food web metric between the boreal and the arctic (Diff B-A) food web and between the arctic II and the arctic (Diff AII-A) food web.

metric	boreal	arctic	arctic II	Diff B-A (%)	Diff AII-A (%)
number of species (S)	180	159	163	12	2
number of links (L)	1546	848	1078	45	21
linkage density (LD)	8.59	5.33	6.61	38	19
connectance (C)	0.05	0.03	0.04	40	25
%-Omni	52	41	43	21	5
%-Can	13	6	8	54	25
% in loops	13	0	3	—	—
meanPath	2.28	2.06	2.05	10	−1
meanOmni	0.40	0.33	0.34	18	3
meanSWTL	2.72	2.61	2.64	4	1
meanClust	0.25	0.17	0.21	32	19
modularity	0.27	0.35	0.30	−30	−17
modularity random ± s.d.	0.19 ± 0.003	0.25 ± 0.004	0.21 ± 0.003	—	—

### (b) Degree distributions and degree centrality maps

**Figure 2.**
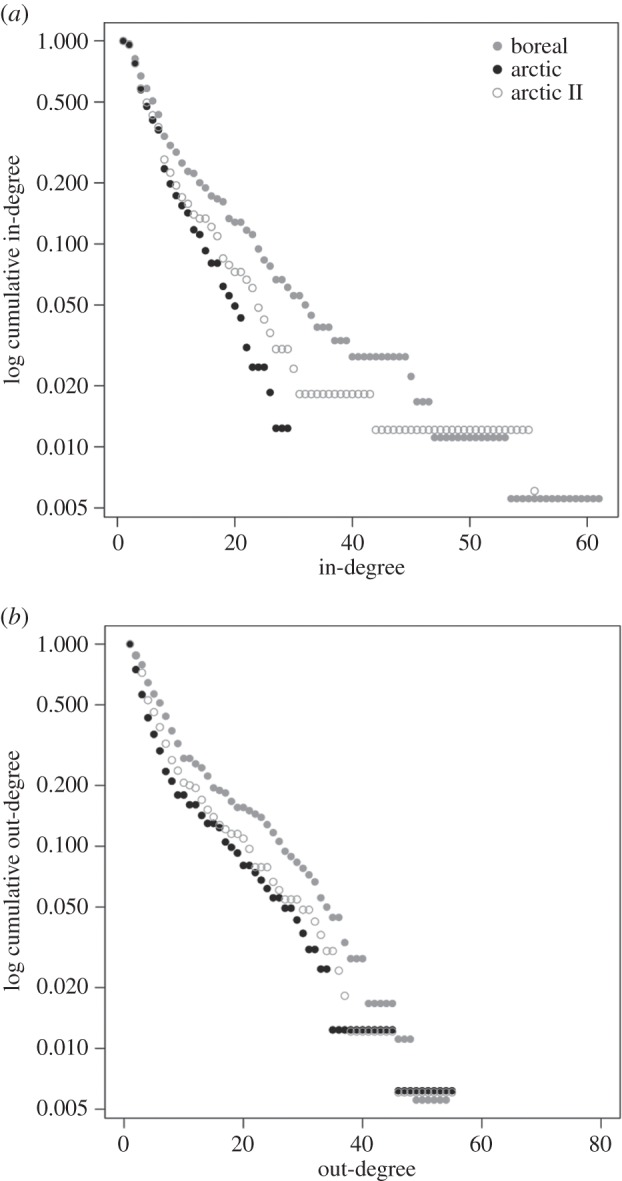
Cumulative (*a*) in- and (*b*) out-degree distributions of the boreal (grey circles), arctic (black circles) and arctic II (open circles) food webs. The in-degree represent the number of prey items of a species, i.e. its generality. The out-degree represent the number of predators of a species, i.e. its vulnerability.

We analysed the in- (number of prey) and out- (number of predators) degree distributions of the food webs (figure 2). The cumulative log-linear plots indicate that in- and out-degree for both regions follow an exponential degree distribution. The boreal food web in-degree distribution was less steep than the arctic, indicating that the boreal food web contained a greater number of trophic generalists. The three trophospecies with the highest degree in the boreal food web were: cod (112 links), haddock (88 links) and beaked redfish (62 links). The three trophospecies with the highest degree in the arctic food web, apart from detritus, were phytoplankton (44 links), polar cod (*Boreogadus saida*, 42 links) and northern shrimp (*Pandalus borealis*, 41 links).

**Figure 3.**
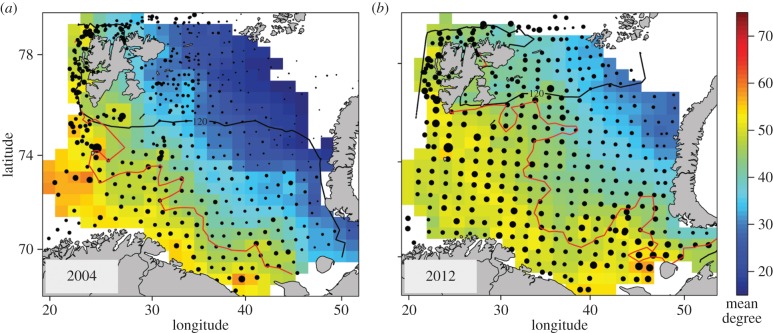
Barents Sea maps of mean degree centrality (mean no. of feeding links) of 51 fish for the years (*a*) 2004 and (*b*) 2012. The dots indicate the position of sampling stations (approx. 400) and the size of the dots is proportional to the mean fish degree at the station. The coloured surface (colour code shown in the legend) indicates the mean degrees of fish, spatially interpolated on a regular grid. North of the 120 day isolines (black lines) sea ice was present for more than 120 days during the year. The red lines indicate the 2°C isoline, southwest of the isoline seawater temperatures are more than 2°C (atlantic water) and northeast of the isoline the temperatures are less than 2°C (mixed water and arctic water). See the electronic supplementary material, figure S2 for the position of the boreal and arctic study regions in the Barents Sea.

After including cod, haddock and the two redfish to the arctic food web, the in-degree distribution of the arctic II food web became less steep. The spatial mappings of the mean degree centrality of fish in the Barents Sea showed clear differences in 2004 between the boreal region characterized by a high mean degree of fish, and the arctic region characterized by a low mean degree of fish. By 2012, this gradient had weakened throughout the whole Barents Sea due to a strong increase in the mean degree in the Arctic (figure 3). The annual (2004–2012) degree centrality maps illustrate how the mean degree centrality of fish in the Arctic increased during the recent period of warming in the Barents Sea (electronic supplementary material, figure S4).

### (c) Modularity and the topological role of species

The modularity analysis divided the boreal food web into four distinct modules, the arctic food web into five and the arctic II food web into four (figure 1). The structure of all empirical food webs was significantly (*p* < 0.01) more modular than that of networks with the same degree distributions but random interaction between species (table 1). Modularity was 30% higher in the arctic food web (0.35) than in the boreal (0.27) (table 1). The LDAs significantly discriminated modules by trophic levels and habitat use (boreal *p* < 0.001, and arctic *p* < 0.001), indicating that some modules are dominated by benthic trophospecies and others by pelagic (electronic supplementary material, figure S5). Modules were also separated across trophic levels (electronic supplementary material, figure S5).

**Figure 4.**
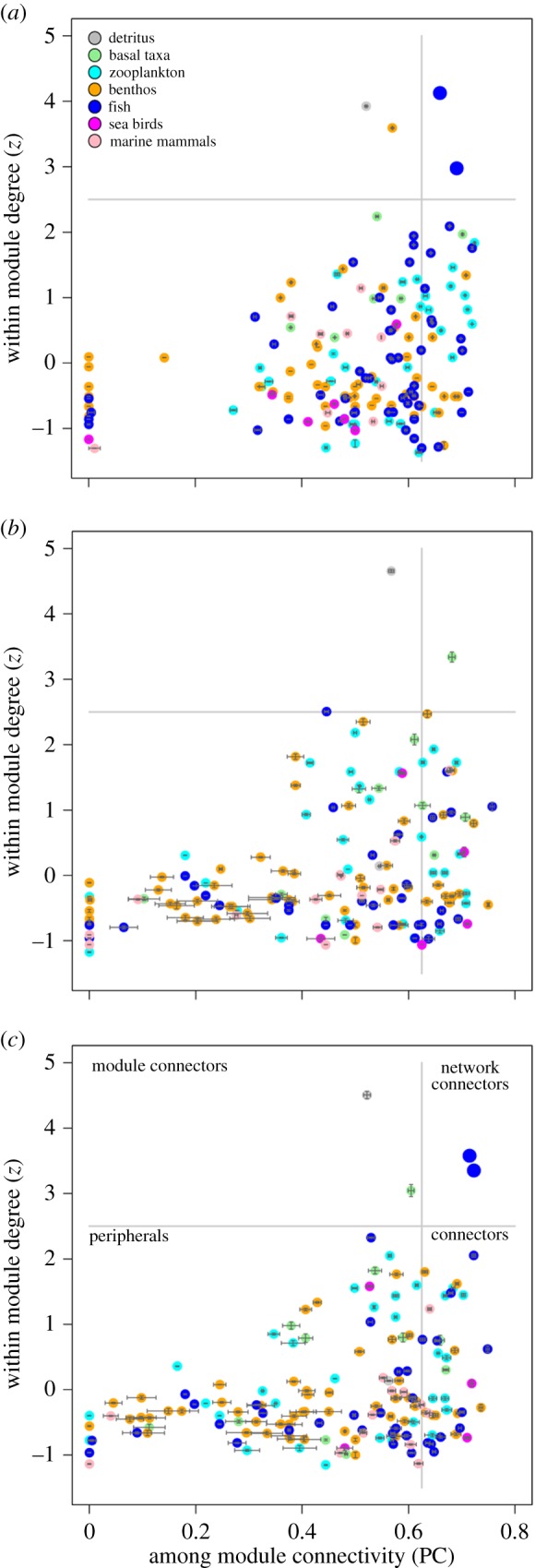
Species topological roles with respect to modularity: (*a*) boreal, (*b*) arctic (*c*) and arctic II food webs. Dots are mean values based on 1000 estimates, and the error bars indicate the 95% CIs for *z* and PC. The topological network roles are: network connectors (upper right), module connectors (upper left), peripherals (lower left) and connectors (lower right). The colours, top left legend in (*a*), correspond to the functional affiliation of a trophospecies: grey, detritus; green, basal taxa; cyan, zooplankton; orange, benthos; blue, fish; magenta, sea birds; light pink, marine mammals. The larger blue dots in (*a*) and (*c*), upper right quadrant, are the two network connector hubs, cod and haddock. See the electronic supplementary material (tables S1 and S2) for lists of all taxa and their topological role.

Topological role analysis showed that, when present, cod and haddock are hub network connectors, playing a high within as well as between-module connecting role (figure 4*a,c*). These two fish are characterized by a high in-degree (many prey) and thus function as super-generalists in the food web. When present in the arctic food web, cod, haddock and beaked redfish (also a module connector) tie modules together, thereby reducing the modularity (table 1 and electronic supplementary material, figure S6). As cod and haddock are the only super-generalists in the food webs and display the most pronounced effect on food web structure, much of the discussion will focus on their food web structuring role. Exhaustive lists of all species and their role as peripherals, connectors, module connectors or network connectors can be found in the electronic supplementary material, tables S1 and S2.

## 4. Discussion and conclusion

### (a) Topological properties of the boreal and the arctic marine food webs

Comparison of food web properties revealed considerable differences in structure and link configuration between the boreal and the arctic food webs in the Barents Sea, despite similar number of taxa in each functional group (i.e. groups such as benthos and fish). The boreal food web displayed higher diversity of trophospecies, mainly due to higher fish species richness, and considerably more links, more cannibals, higher clustering and more trophospecies in loops. The greater number of feeding links in the boreal food web is due to more generalists at higher trophic levels. The presence of relatively many species in loops in the boreal food web may be attributed to higher mutual predation and higher levels of omnivory, whereas higher clustering may be attributed to a greater incidence of within-chain omnivory [39].

The two food webs also displayed similarities, e.g. they had similar mean path lengths and mean trophic levels. The high number of omnivores and cannibals seems to be an inherent feature of marine systems when compared to non-marine systems [32], probably because many fish commonly use these feeding strategies [40]. The food webs had short path length (two degrees of separation); a seemingly universal property of food webs, indicating that species are close neighbours [41,42]. This implies that environmental perturbations, e.g. from climate warming or overfishing, can spread rapidly through the food web, affecting many species indirectly. Such indirect, higher-order effects of environmental perturbations, i.e. effects mediated via a third species by predation or competition, may have greater impact on food web configuration than direct effects [43]. Recent studies from arctic terrestrial and marine systems show how climate-driven effects on community structure are often indirect and mediated via predation or facilitation [4,44].

### (b) Degree distributions and degree centrality maps

The estimated degree distributions follow an exponential distribution, which supports previous studies of food webs with mid-range (0.03–0.05) connectance [41]. However, the in-degree (no. of prey) distribution was steeper for the arctic food web, suggesting that, on average, arctic trophospecies have narrower niches than boreal trophospecies. The arctic marine food web is known to contain many specialized benthivore fish (e.g. *Liparis* spp.) [5], but also many sympagic specialists (e.g. polar cod and the crustacean *Gammarus wilkitzkii*) which live in close association with the sea ice habitat. On the other hand, the boreal marine region of the Barents Sea is characterized by many large generalists as documented in this study (e.g. cod, haddock, wolffishes *Anarhichas* spp., redfishes, etc.).

The mean degree centrality maps show that fish in the southern Barents Sea have, on average, a higher degree. In 2004, the contrast between the mean degree in the boreal and the arctic region was sharp, but by 2012 this difference had weakened because of the increased mean degree of fish in the Arctic. The extensive spatial changes in the mean degree of fish in the period 2004–2012 (illustrated by our degree centrality maps) highlight the rapid structural changes taking place in the arctic Barents Sea due to the poleward shifts of boreal generalists. The recent increase of boreal fish generalists in the Arctic can be explained by their ability to take advantage of a diverse range of prey and adjust to a varying and unpredictable environment as experienced in a warming Arctic [45]. A property such as generalism directly affects the interaction structure of species and may have many indirect high-order effects on food web structure (e.g. by connecting energetic pathways and changing interaction parameters between species). The increase of fish diversity and abundances at higher trophic levels in the arctic region of the Barents Sea could enhance top-down regulation of the arctic marine food web [5,29].

### (c) Modularity and topological roles of trophospecies

One of the food web properties that is strongly affected by highly connected generalists is modularity. The importance of highly connected nodes, network hubs, for modularity has been stressed previously in a variety of biological systems, including genetic [46], metabolic [12], spatial [19,47,48], mutualistic [49,50] and food web networks [21]. We show that, in the Arctic, inclusion of the boreal super-generalists leads to a decrease in modularity. The extent of this decrease depends on the nature of the modules and on how the species' links are distributed within and among modules, i.e. a species' topological role. In this study, food web modules are significantly associated with habitats (benthic and pelagic) and trophic levels, stressing that habitats form natural boundaries for marine food web modules, a result consistent with previous findings from a Caribbean marine ecosystem [21].

Given that modules are separated by habitats, habitat generalists like cod will forage across modules, linking modules and reducing overall modularity. Two of the poleward moving species, cod and haddock, have the widest ecological niches in our study, being generalists and omnivores. By feeding across many trophic levels and across pelagic and benthic habitats, these species have a particularly strong effect on modularity. The increased coupling of benthic and pelagic habitats by these fish will potentially lead to changes in ecosystem functioning in the Arctic. We hypothesize that energetic pathways across modules will increase, promoting the transfer of matter and energy from one module to another, as well as the spread and effects of perturbations.

From another well-studied marine region (Nova Scotia) in the northwest Atlantic, we know that the sudden disappearance of cod has led to the reorganization of the food web [51]. The removal of cod was followed by an increase in benthic crustaceans such as northern shrimp and snow crab (*Chionoecetes opilio*). Northern shrimp and the invasive snow crab are present in the northeast Barents Sea food web, where they play a module connecting role. We conjecture here that the abrupt structural shift induced by cod in Nova Scotia may be attributed to cod's role as a food web network connector hub. The take-over by northern shrimp and snow crab could indicate that the loss of an important network connector may be substituted by increasing abundances of other module connecting species, performing similar module connecting roles, but changing community structure fundamentally due to distinct trophic network positions.

Species with fewer interactions may also have large structural impact depending on their network position (and on the strength of their interactions) [14,18]. Our topological role analysis shows that a few species (approx. 20%) are structurally very important. These trophospecies connect modules, but not the entire network. Module connectors in the Barents Sea food webs are key species occupying high trophic levels such as beaked redfish, polar cod, wolffishes, snow crab, fulmar (*Fulmarus glacialis*), ringed and bearded seal (*Phoca hispida* and *Erignathus barbatus*), but also low trophic level species such as northern shrimp, northern krill (*Meganyctiphanes norvegica*) and calanoid copepods (*Calanus* spp.). Interestingly, several taxa with few trophic interactions (low degree) possess module connecting roles due to their position in the network, e.g. the eelpout (*Lycodes gracilis*) and the sculpin species (*Triglops* spp. and *Icelus* spp.). Populations of sculpins (*Icelus* spp.) have been declining recently in the arctic region of the Barents Sea, which is remarkable considering that the arctic region has become more productive [5,28]. A potential explanation for the recent local decline of these structurally important arctic fish species are predation and competition by the increasingly abundant boreal fish generalists [5].

### (d) Observed and expected changes in arctic marine food web structure

Increasing seawater temperatures, reduced sea ice coverage and longer duration of the ice-free periods will open ‘thermal’ windows of opportunity for expanding boreal species and novel communities in the Arctic [28]. Fish, in particular, but also sea birds are among the quickest to respond to climate warming due to their high motility. In the northern Barents Sea, boreal sea birds (e.g. *Alca torda*, *Fratercula arctica* and *Uria aalge*) are increasing in abundance [52]. These migratory top predators move into suitable habitats in the search for prey, and indeed, in the arctic region of the Barents Sea, they find a pelagic community increasingly dominated by atlantic zooplankton such as *Calanus finmarchicus*, krill and capelin [52]. We hypothesize that the increase in boreal prey availability may favour boreal top predators in the resource competition with arctic top predators.

The observed changes in arctic community structure alter interaction parameters, particularly between taxa at higher trophic levels [53]. While many boreal taxa have become more abundant in the northern Barents Sea, some arctic taxa have been declining. For example, abundances of the arctic sea birds, Brünnichs guillemot (*Uria lomvia*) and possibly little auk (*Alle alle*) are declining, as is the case for some arctic pelagic (e.g. polar cod) and benthivore fish (e.g. *Liparis* spp.) [5,52]. Marine mammals (the harp seal *Phagophilus groenlandicus* and the minke whale *Balenoptera acutorostrata*) also seem to be affected by the presence of boreal fish competitors (e.g. cod) as indicated by observed declines in their body condition [53]. Although it is notoriously hard to predict the outcome of species interactions, the effects of alterations in energetic pathways within and between pelagic and benthic compartments of the arctic food web will have far-reaching ramifications for dynamics and functioning, permeating through the entire food web network.

## 5. Concluding remarks

Some of the most prompt responses to climate warming are altered migration patterns of opportunistic, generalist fish. The poleward expansion of these fish generalists alters the structure of arctic food webs, increasing the connectivity between benthic and pelagic habitat modules while reducing the modularity. Establishing and reinforcing energetic pathways between food web compartments will affect ecosystem functioning. We expect that in a more densely connected and less modular arctic marine food web, species will be closer neighbours, resulting in matter, energy, but also the effects of perturbations, spreading further and faster across the ecosystem.

## Supplementary Material

Electronic supporting material ID RSPB-2015-1546.pdf

## Data accessibility

The food web data used in this study can be downloaded from the Dryad repository: http://dx.doi.org/10.5061/dryad.73r6j.
