# Supplementary material for: Climate change alters the structure of arctic marine food webs due to poleward shifts of boreal generalists
Source: Proc Biol Sci. 2015 Sep 7;282(1814):20151546. doi: 10.1098/rspb.2015.1546 (PMC4571709; doi:10.1098/rspb.2015.1546)
Supplement: Electronic supporting material ID RSPB-2015-1546.pdf [file rspb20151546supp1.pdf]

Electronic supporting material

Climate change alters the structure of Arctic marine food webs due to poleward shifts of boreal generalists

Susanne Kortsch, Raul Primicerio, Maria Fossheim, Andrey V. Dolgov, and Michaela Aschan

Appendix S1. Detailed description of the study regions and subsampling of the marine food webs

(a) *Barents Sea*

The Barents Sea is an open arcto-boreal shelf sea and is the largest marginal shelf sea in the Arctic Ocean (figure S1). The Barents Sea ecosystem is profoundly influenced by the inflow of relatively warm and saline atlantic water (T>3°C, S >35 ‰) in the south-west, and arctic water masses (T<0°C, 34.3 ‰ ≤ S ≤ 34.8 ‰) in the North. The mixing of atlantic and arctic water masses at the polar front, as well as the presence of the marginal ice zone, strongly influence biological production. The Barents Sea is one of the most productive marine ecosystems in the world [1]. In the Arctic, the Barents Sea accounts for 49 % of total pan-arctic shelf primary production and supports some of the largest fish stocks of the world including north-east arctic cod (*Gadus morhua*), polar cod (*Boreogadus saida*), capelin (*Mallotus villosus*) and herring (*Clupea harengus*) [2, 3]. Due to easy access, high productivity and its importance for fisheries, the Barents Sea is one of the best studied marine ecosystems in the Arctic [4]. Since 2004, Norwegian and Russian research institutions have sampled taxa from the entire food web in a joint effort to map the whole ecosystem [5].

(b) ***Boreal and arctic study regions of the Barents Sea***

We have defined the boreal (186 400 km^2) and arctic (304 067 km^2) study regions based on hydrology and species distributions (figure S2a). To capture a good snapshot representation of a boreal and an arctic food webs of the Barents Sea, we chose areas south-west and north-east of the polar front, which is the main hydrological demarcation separating boreal and arctic regions of the Barents Sea (figure S2b). The exact position of the polar front varies from year to year and is most variable in the east depending on the strength of the atlantic water inflow [6]. The past decade (2000-2010) has been the warmest on record and warmer and more salty atlantic water masses have extended further north and north-east into the Barents Sea resulting in a contraction of the area covered by arctic water masses [7, 8]. In addition to hydrological data, we used fish abundance data to define the position of the two regions [9].

(c) ***Occurrence of fish and epibenthos***

In this study, presence and absence of fish was integrated over the early sampling period 2004-2007 to specify occurrence of species within study areas. The early years of the sampling were chosen in order to capture a snapshot of the arctic food web prior to the pronounced distributional shifts of large fish driven by rapid warming [2, 10]. After 2007, the surveys reveal how boreal fish are moving even further north-east into the arctic regions of the Barents Sea [2,11]. Data on the occurrence of epibenthos are from 2011. To overcome the problem of including rare and only occasionally sampled fish and epibenthos, we used distribution maps and additional abundance information for sub-sampling among these groups (figure S2b). In the boreal study region, fish were sampled at 308 stations (2004-2007) and epibenthos at 38 stations (2011). In the arctic study region, fish were sampled at 327 stations and epibenthos at 50 (figure S1b). Three selection criteria were formulated for each

trophospecies: 1) mean abundance of a taxon within study areas; 2) max abundance of a taxon within study areas; and 3) proportion of stations in which a taxon was found. A trophospecies was included in the food web of a given area if it was selected based on at least one of the three selection criteria. In some cases, the quantitative selection criteria had to be complemented with knowledge of species spatial distribution prior to 2004. For example, we chose not to include cod and haddock in the arctic food web because these two taxa where hardly present in the arctic study region prior to warming. Likewise, we excluded polar cod from the boreal food webs, although a few individuals can sometimes be caught in the boreal region of the Barents Sea.

(d) *Food web data: shortcomings and strengths*

Trophospecies included in the food web database (meta-web) were selected according to at least one of the following criteria: 1) abundance, 2) spatial distribution and 3) existing knowledge of trophic relationships. Rare species were not included. In our food webs, individual trophospecies usually correspond to taxonomic species, but can sometimes refer to higher taxonomic groups, e.g. genus, family, and class. Other large aggregations include the basal taxa such as phytoplankton, diatoms, heterotroph flagellates or bryozoans. While compiling the database, we made considerable efforts to obtain a balanced representation of the different functional groups and their feeding links. Yet, we are aware that, for some of the arctic taxa, research and literature is sparser and this may have led to an underestimation of their feeding links.

To date, most marine food webs are systematically biased towards higher trophic levels because of incomplete diet information and poor sampling of basal species. We acknowledge that a better resolution at the basal level would increase the percentage of low trophic level

species and decrease the relative proportion of intermediate species. Expansion of the basal compartment (particularly phytoplankton and benthos) should be prioritized to reduce this bias and to improve the representation of the complexity in future marine food webs. Although historically food webs have under-represented the number of species and their links [12], recent compilations of food webs are becoming increasingly comprehensive, with higher resolution, level of detail and scale [13]. Our Barents Sea food webs are examples of highly resolved ecological networks, and unlike most comparative food web studies, the level of trophospecies aggregation between the study regions is identical. This implies that dissimilarities observed between Boreal and Arctic food webs here cannot be attributed to differences in the classification and aggregation of trophospecies, but must be attributed to differences in species composition and link configuration of the regional food webs.

The dataset file of the meta-web for the whole Barents Sea can be downloaded from the *Ecological archives* website [5], while the boreal and the arctic Barents Sea food web files of the present study can be downloaded from the Dryad repository (doi:10.5061/dryad.73r6j).

References

1. Carmack, E., Wassmann, P., 2006 Food webs and physical-biological coupling on pan-Arctic shelves: unifying concepts and comprehensive perspectives. *Progr. Oceanogr.* **71**, 446-477. (doi:10.1016/j.pocean.2006.10.004)
2. Michalsen, K., Dalpadado, P., Eriksen, E., Gjøsæter, H., Ingvaldsen, R. B., Johannesen, E., Jørgensen, L. L., Knutsen, T., Prozorkevich, D., Skern-Mauritzen, M. 2013 Marine living resources of the Barents Sea–Ecosystem understanding and monitoring in a climate change perspective. *Mar. Biol. Res.* **9**, 932-947. (doi:10.1080/17451000.2013.775459)
3. Sakshaug, E., Johnsen, G. H., Kovacs, K. M. 2009 *Ecosystem Barents Sea*, Tapir Academic Press. pp. 57-81.

4. Wassmann, P., Duarte, C. M., Agusti, S., Sejr, M. K. 2011 Footprints of climate change in the Arctic marine ecosystem. *Glob. Change Biol.* **17** (2), 1235-1249. (doi:10.1111/j.1365-2486.2010.02311.x)
5. Planque, B., Primicerio, R., Michalsen, K., Aschan, M., Certain, G., Dalpadado, P., Gjøsæater, H., Hansen, C., Johannesen, E., Jørgensen, L.L., Kolsum, I,. Kortsch, S., Leclerc, L-M., Omli, L., Skern-Mauritzen, M., Wiedmann, M. 2014 Who eats whom in the Barents Sea: a food web topology from plankton to whales: Ecological Archives E095-124. *Ecology* **95**, 1430-1430. (doi:10.1890/13-1062.1)
6. Loeng, H., Drinkwater, K. 2007 An overview of the ecosystems of the Barents and Norwegian Seas and their response to climate variability. *Deep Sea Res. II.* **54**, 2478-2500. (doi:10.1016/j.dsr2.2007.08.013)
7. Smedsrud, L.H., Ingvaldsen, R., Nilsen, J., Skagseth, Ø. 2010 Heat in the Barents Sea: transport, storage, and surface fluxes. *Ocean Sci.* **6**, 219-234. (doi:10.5194/os-6-219-2010)
8. Ellingsen, I. H., Dalpadado, P., Slagstad, D., Loeng, H. 2008 Impact of climatic change on the biological production in the Barents Sea. *Clim. change* **87**, 155-175. (doi:
10.1007/s10584-007-9369-6)
9. Fossheim M., Primicerio, R., Johannesen, E., Ingvaldsen, R. B., Aschan, M. M., Dolgov, A. V. 2015 Recent warming leads to a rapid borealization of fish communities in the Arctic. *Nature Clim. Change* (doi:10.1038/nclimate2647)
10. Wiedmann, M. A., Aschan, M., Certain, G., Dolgov, A., Greenacre, M., Johannesen, E., Planque, B., Primicerio, R. 2014 Functional diversity of the Barents Sea fish community. *Mar. Ecol. Prog. Ser.* **495**, 205-218. (doi:103354/meps10558)
11. Hollowed, A.B., Sundby, S. 2014 Change is coming to the northern oceans. *Science* **344**, 1084-1085. (doi: DOI: 10.1126/science.1251166)
12. Polis, G. A. 1991 Complex trophic interactions in deserts: an empirical critique of food-web theory. *Am. Nat.*123-155.
13. Dunne, J. A., Lafferty, K. D., Dobson A. P., Hechinger, R. F., Kuris, A. M., Martinez, N. D., McLaughlin, J. P., Mouritsen, K. N., Poulin, R., Reise, K. 2013 Parasites affect food web structure primarily through increased diversity and complexity. *PLoS biol.* **11**, e1001579. (doi: 10.1371/journal.pbio.1001579)

Appendix S2. Supplementary figures and tables

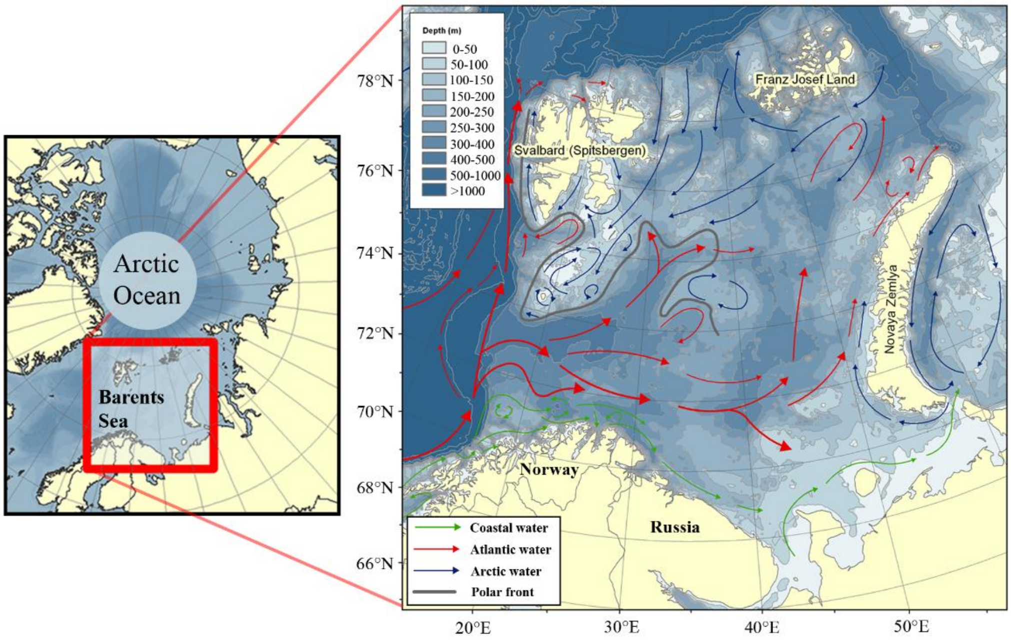

Figure S1. Map of the Barents Sea (to the right) and of its location (to the left) in the Arctic. The topography is indicated with the blue gradients, see legend in the top-left corner. The atlantic (red) and arctic (blue) ocean currents within the Barents Sea are indicated with arrows, see legend in the bottom-left corner.

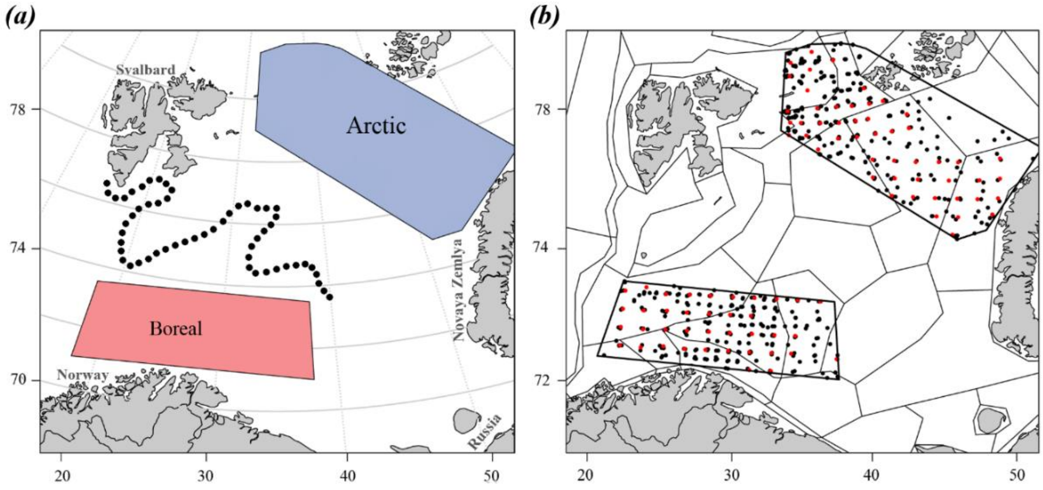

Figure S2. *(a)* Location of the boreal (red) and arctic (blue) study regions within the Barents Sea. The black dotted line indicates the approximate position of the polar front, separating atlantic and arctic water masses. *(b)* Location of the sampling stations for fish (black dots) and epibenthos (red dots) within the boreal and the arctic study regions used to subsample the boreal and the arctic food webs. The remaining trophospecies (basal taxa, zooplankton, benthic infauna, sea birds and marine mammals) were assigned to the study regions via their presence/absence within the indicated polygons.

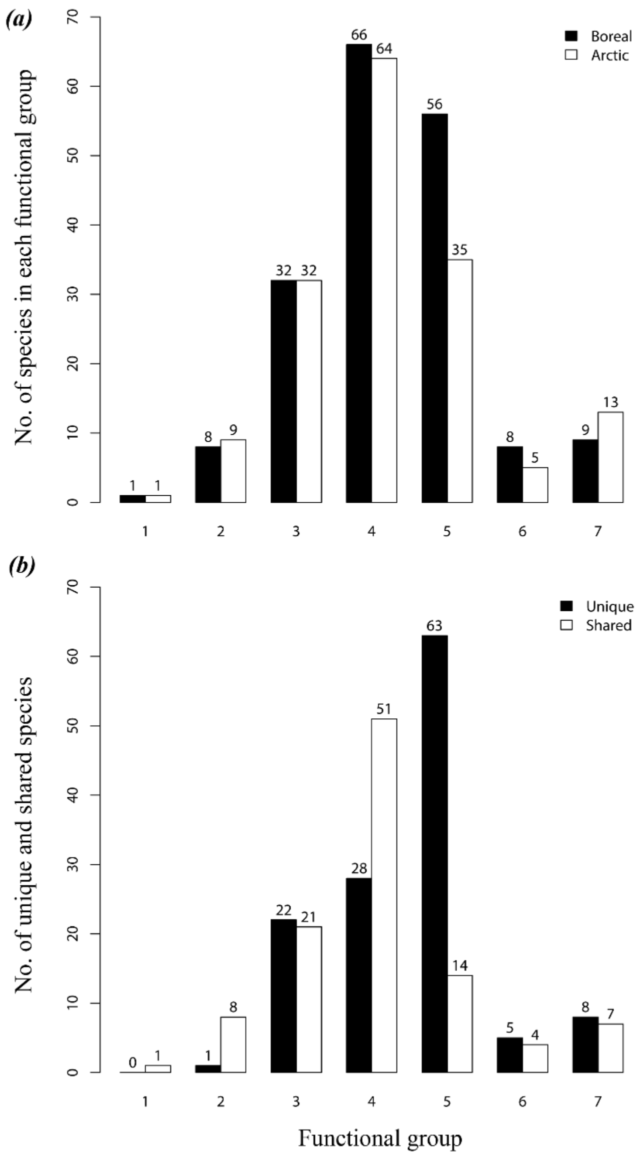

Figure S3. *(a)* Number of trophospecies in each functional group of the boreal (black bars) and arctic (white bars) food webs. *(b)* Number of unique (black bars) and shared (white bars) trophospecies between the boreal and arctic food web. The functional groups are: 1=detritus; 2=basal taxa; 3=zooplankton; 4=benthos; 5=fish; 6=sea birds; 7=marine mammals.

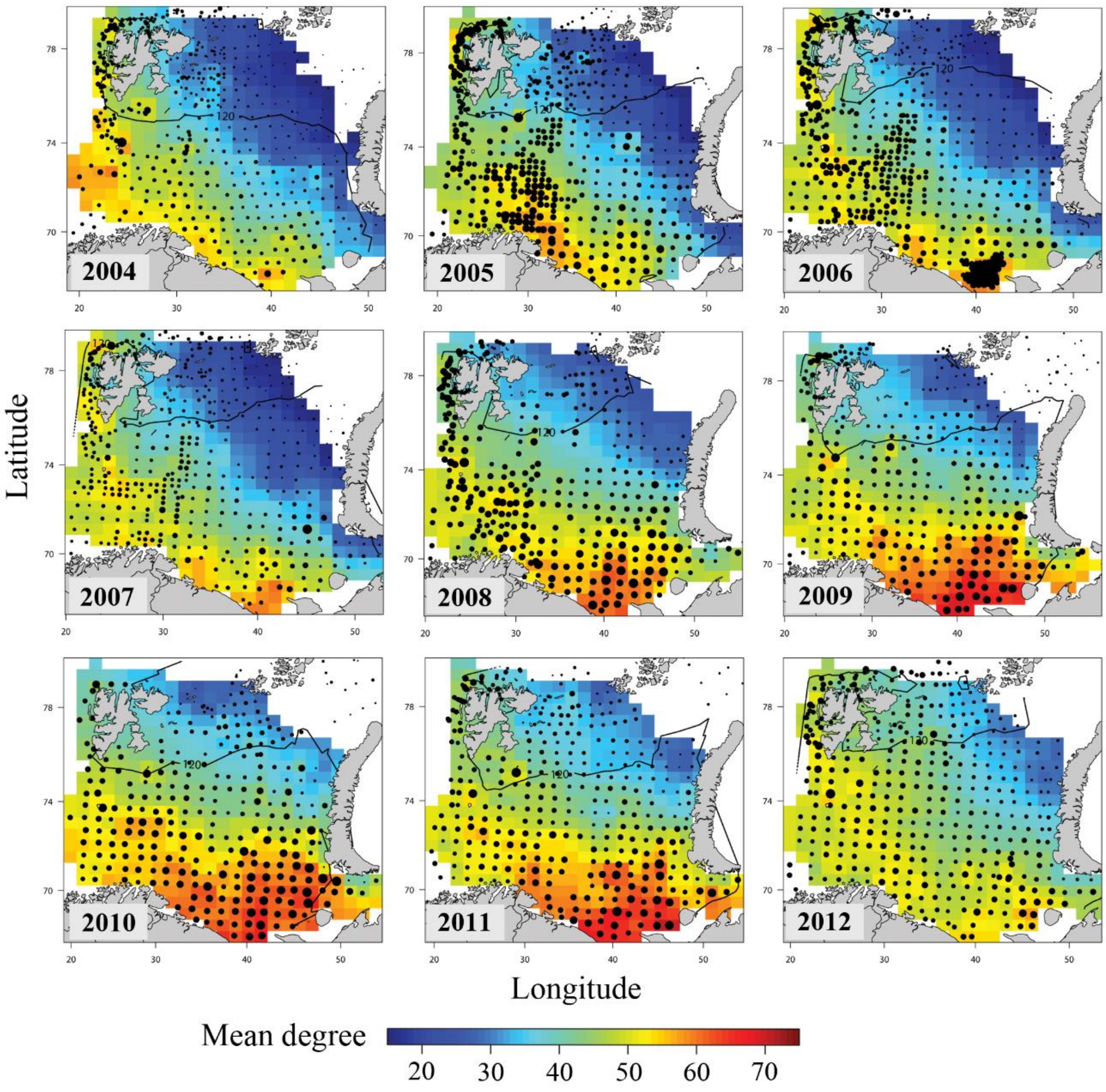

Figure S4. Barents Sea maps of the yearly mean degree centrality (number of feeding links) of fish during the time period 2004 to 2012. The dots indicate the position of sampling stations (~400) and the size of the dots is proportional to the mean fish degree at the station. The coloured surface (colour code shown in the legend) indicates the mean degree of fish spatially interpolated on a regular grid. North of the 120 day isolines (black lines) sea ice was present for more than 120 days during the year.

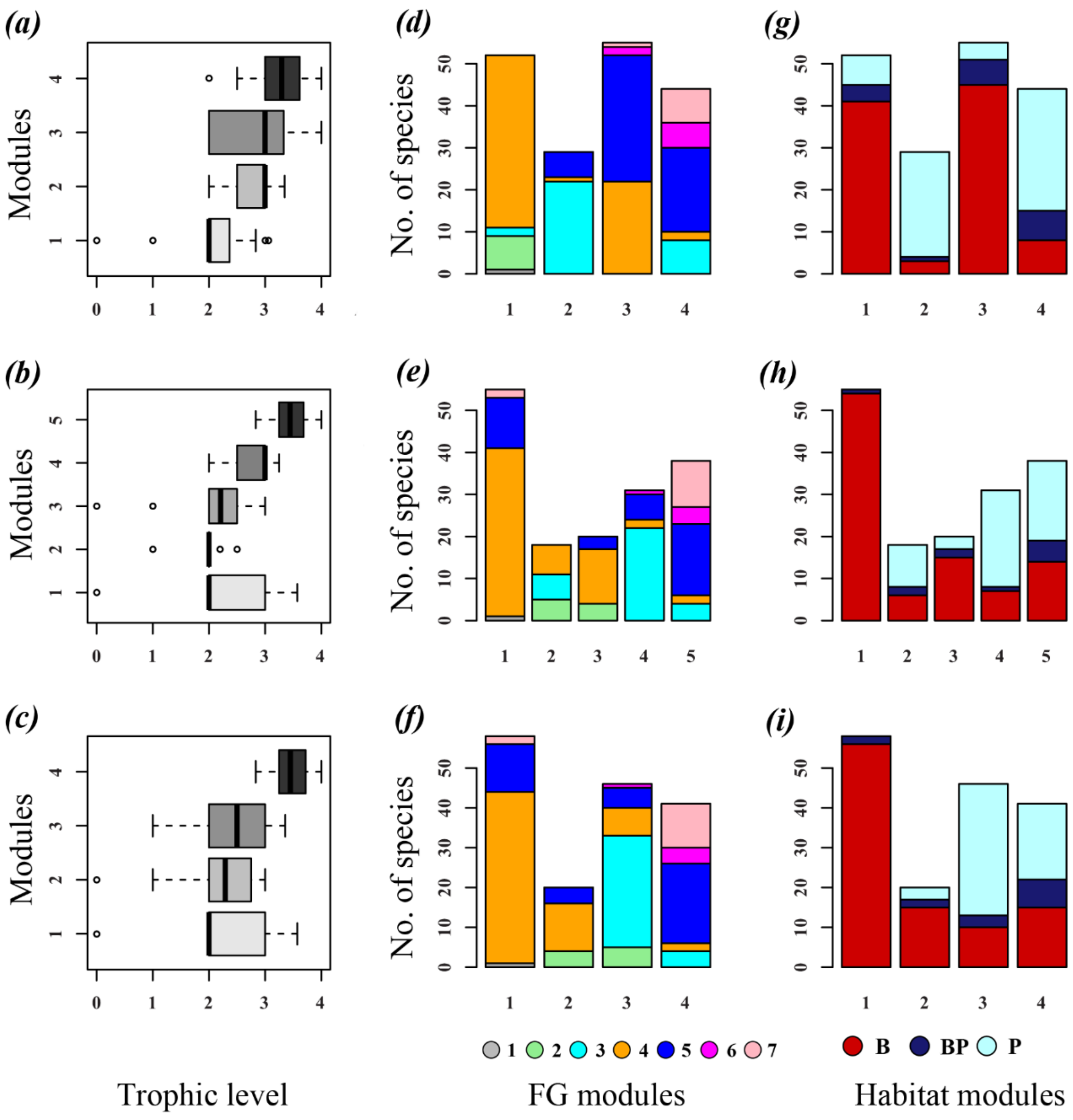

Figure S5. Boxplot of the trophic level of trophospecies within each module in *(a)* the boreal, *(b)* the arctic and *(c)* the arctic II food webs. Bar plot of the number of species within each module of *(d)* the boreal, *(e)* the arctic, *(f)* and the arctic II food webs. The colours in the bar plots indicate the functional group (FG) affiliation: grey=detritus (1); green=basal taxa (2); cyan=zooplankton (3); orange=benthos (4); blue=fish (5); magenta=sea birds (6); light pink=marine mammals (7). Bar plots of *(g)* the boreal, *(h)* the arctic, and *(i)* the arctic II food web showing the frequency of benthic=red, bentho-pelagic=dark-blue and pelagic=light-blue trophospecies within each module.

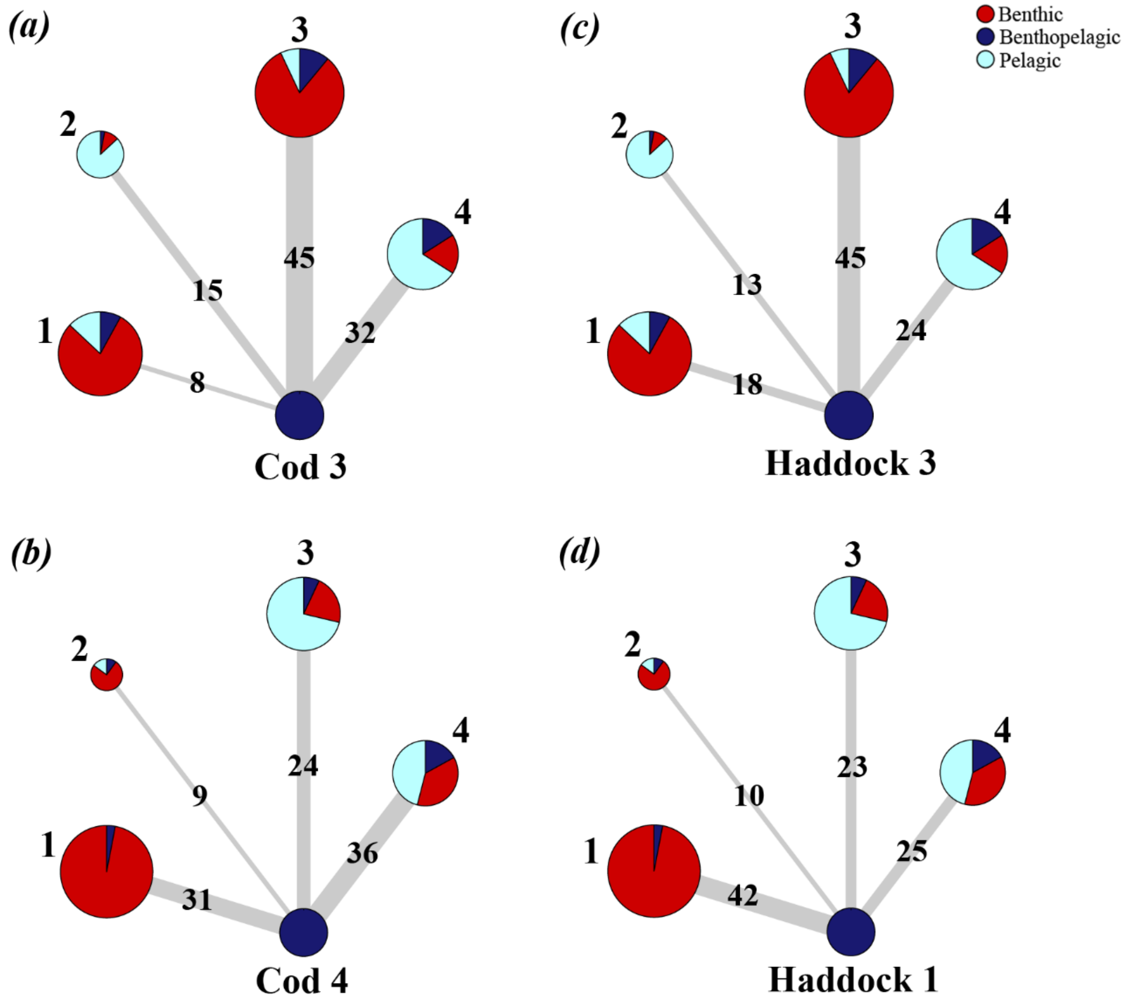

Figure S6. The module affiliation of the two network hubs, cod and haddock, and their distribution of links to species within their own module and to species in other modules for the boreal and the arctic II food web. Linkage structure of *(a)* cod in the boreal food web, *(b)* cod in the arctic II food web, *(c)* haddock in the boreal food web, and *(d)* haddock in the arctic II food web. The number next to the fish name indicates which module cod and haddock belong to. The number on the edges (grey lines) indicate how many links cod and haddock have to species in these modules. Bi-directional links (mutual predation links) are only counted once. The colored pie-charts show the proportion of pelagic (light blue), benthic (red) and bentho-pelagic (blue) species within each module.

Table S1. List of taxa (in alphabetical order within each functional group) included in the boreal region of the Barents Sea, and their functional affiliation, habitat use, degree (number of trophic interactions) and topological role.

Index	Taxon	Functional group	Habitat	Degree	Topological role
1	Detritus	1 Detritus	Benthopelagic	53	Module hub
2	Autothroph_flagellat	2 Basal taxa	Pelagic	22	Peripheral
3	Bacteria_indet	2 Basal taxa	Benthopelagic	13	Peripheral
4	Diatom	2 Basal taxa	Benthopelagic	33	Peripheral
5	Heterotroph_flagellat	2 Basal taxa	Pelagic	21	Peripheral
6	Macroalgae	2 Basal taxa	Benthic	4	Peripheral
7	Mixotroph_flagellates	2 Basal taxa	Pelagic	5	Peripheral
8	Phytoplankton_indet	2 Basal taxa	Pelagic	47	Module connector
9	Protozooplankton	2 Basal taxa	Pelagic	13	Peripheral
10	*Acartia_spp*	3 Zooplankton	Pelagic	18	Module connector
11	*Aglantha_digitale*	3 Zooplankton	Pelagic	6	Peripheral
12	*Aurelia_aurita*	3 Zooplankton	Pelagic	12	Peripheral
13	*Beroë_sp*	3 Zooplankton	Pelagic	14	Peripheral
14	*Bolinopsis_infundibulum*	3 Zooplankton	Pelagic	14	Peripheral
15	*Calanus_finmarchicus*	3 Zooplankton	Pelagic	52	Module connector
16	*Calanus_glacialis*	3 Zooplankton	Pelagic	39	Module connector
17	*Calanus_hyperboreus*	3 Zooplankton	Pelagic	43	Module connector
18	*Clione_limacina*	3 Zooplankton	Pelagic	9	Peripheral
19	*Cyanea_capillata*	3 Zooplankton	Pelagic	21	Peripheral
20	*Dimophyes_arctica*	3 Zooplankton	Pelagic	2	Peripheral
21	*Eukrohnia_hamata*	3 Zooplankton	Pelagic	10	Peripheral
22	*Fritillaria_borealis*	3 Zooplankton	Pelagic	4	Peripheral
23	*Limacina_helicina*	3 Zooplankton	Pelagic	12	Peripheral
24	*Limacina_retroversa*	3 Zooplankton	Pelagic	12	Peripheral
25	*Meganyctiphanes_norvegica*	3 Zooplankton	Pelagic	42	Module connector
26	*Metridia_longa*	3 Zooplankton	Pelagic	26	Module connector
27	*Nematoscelis_megalops*	3 Zooplankton	Pelagic	3	Peripheral
28	*Oikopleura_dioica*	3 Zooplankton	Pelagic	5	Peripheral
29	*Oikopleura*_spp	3 Zooplankton	Pelagic	12	Module connector
30	*Oithona_similis*	3 Zooplankton	Pelagic	6	Peripheral
31	*Oithona_spinirostris/atlantica*	3 Zooplankton	Pelagic	26	Module connector
32	*Oncaea_borealis*	3 Zooplankton	Pelagic	9	Peripheral
33	*Pareuchaeta_norvegica*	3 Zooplankton	Pelagic	11	Peripheral
34	*Pareuchaeta*_spp	3 Zooplankton	Pelagic	9	Peripheral
35	*Pseudocalanus*_spp	3 Zooplankton	Pelagic	24	Peripheral
36	*Sagitta*_spp	3 Zooplankton	Pelagic	33	Module connector
37	*Sarsia*_spp	3 Zooplankton	Pelagic	6	Peripheral
38	*Themisto_abyssorum*	3 Zooplankton	Pelagic	32	Module connector
39	*Thysanoessa_inermis*	3 Zooplankton	Pelagic	45	Module connector
40	*Thysanoessa_longicaudata*	3 Zooplankton	Pelagic	33	Peripheral
41	*Thysanoessa_raschii*	3 Zooplankton	Pelagic	38	Peripheral
42	Actiniaria_g_sp	4 Benthos	Benthic	11	Module connector

43	*Aglaophamus_malmgreni*	4 Benthos	Benthic	6	Peripheral
44	*Aphelochaeta_marioni*	4 Benthos	Benthic	6	Peripheral
45	*Arrhis_phyllonyx*	4 Benthos	Benthic	8	Module connector
46	Ascidiacea_g_sp	4 Benthos	Benthic	6	Peripheral
47	Asellota_indet	4 Benthos	Benthic	7	Peripheral
48	*Astarte*_sp	4 Benthos	Benthic	10	Peripheral
49	*Asterias_rubens*	4 Benthos	Benthic	13	Peripheral
50	*Balanus*_sp	4 Benthos	Benthic	11	Peripheral
51	*Bathyarca_galacialis*	4 Benthos	Benthic	3	Module connector
52	Bryozoa_indet	4 Benthos	Benthic	4	Peripheral
53	Benthos_larvae	4 Benthos	Benthopelagic	8	Module connector
54	*Buccinum*_sp	4 Benthos	Benthic	9	Peripheral
55	*Chaetozone*_sp	4 Benthos	Benthic	5	Peripheral
56	*Chlamys_islandica*	4 Benthos	Benthic	7	Peripheral
57	*Chone*_sp	4 Benthos	Benthic	4	Peripheral
58	*Ciona_intestinalis*	4 Benthos	Benthic	6	Peripheral
59	Cirratulidae_indet	4 Benthos	Benthic	6	Peripheral
60	*Colus*_sp	4 Benthos	Benthic	4	Peripheral
61	*Ctenodiscus_crispatus*	4 Benthos	Benthic	8	Peripheral
62	*Cucumaria_frondosa*	4 Benthos	Benthic	9	Peripheral
63	*Erythrops*_sp	4 Benthos	Benthic	12	Peripheral
64	Euclymeninae_indet	4 Benthos	Benthic	3	Peripheral
65	Foraminifera	4 Benthos	Benthic	16	Peripheral
66	*Galathowenia*_sp	4 Benthos	Benthic	3	Peripheral
67	Gammaridae_indet	4 Benthos	Benthic	32	Peripheral
68	*Geodia*_sp	4 Benthos	Benthic	3	Peripheral
69	*Gonatus_fabricii*	4 Benthos	Pelagic	33	Peripheral
70	*Heteromastus_filiformis*	4 Benthos	Benthic	4	Peripheral
71	*Hyas*_sp	4 Benthos	Benthic	12	Peripheral
72	Hydrozoa_indet	4 Benthos	Benthic	1	Peripheral
73	*Lumbriclymene_minor*	4 Benthos	Benthic	7	Peripheral
74	*Lumbrineris*_sp	4 Benthos	Benthic	18	Peripheral
75	*Macoma*_sp	4 Benthos	Benthic	9	Peripheral
76	*Maldane_sarsi*	4 Benthos	Benthic	5	Peripheral
77	*Mendicula_ferruginosa*	4 Benthos	Benthic	4	Module connector
78	*Molpadia_borealis*	4 Benthos	Benthic	11	Peripheral
79	*Myriochele_herri*	4 Benthos	Benthic	1	Peripheral
80	*Nephtheidae*_sp	4 Benthos	Benthic	3	Peripheral
81	*Nyctiphanes_couchii*	4 Benthos	Benthic	7	Module connector
82	*Ophiopholis_aculeata*	4 Benthos	Benthic	19	Peripheral
83	*Ophiura*_sp	4 Benthos	Benthic	30	Peripheral
84	Ostracoda_indet	4 Benthos	Benthic	22	Module connector
85	*Pagurus*_sp	4 Benthos	Benthic	15	Peripheral
86	*Pandalus_borealis*	4 Benthos	Benthic	59	Module connector
87	*Paralithodes_camtschaticus*	4 Benthos	Benthic	55	Module hub
88	*Paramphinome_jeffreysii*	4 Benthos	Benthic	3	Peripheral

89	*Phascolion_strombus*	4 Benthos	Benthic	3	Peripheral
90	Polychaeta	4 Benthos	Benthic	36	Peripheral
91	Polynoidae_indet	4 Benthos	Benthic	17	Peripheral
92	*Pontaster_tenuispinus*	4 Benthos	Benthic	3	Peripheral
93	*Pontophilus_norvegicus*	4 Benthos	Benthic	10	Peripheral
94	Porifera_g_sp	4 Benthos	Benthic	8	Peripheral
95	*Prionospio_cirrifera*	4 Benthos	Benthic	4	Peripheral
96	*Rhachotropis_macropus*	4 Benthos	Benthic	9	Module connector
97	*Rossia*_sp	4 Benthos	Benthic	15	Peripheral
98	*Sabinea*_sp	4 Benthos	Benthic	17	Peripheral
99	*Scalibregma_inflatum*	4 Benthos	Benthic	6	Peripheral
100	*Similipecten_greenlandicus*	4 Benthos	Benthic	5	Peripheral
101	*Spiophanes_kroeyeri*	4 Benthos	Benthic	4	Peripheral
102	Spirorbidae_indet	4 Benthos	Benthic	2	Peripheral
103	*Stichopus_tremulus*	4 Benthos	Benthic	8	Peripheral
104	*Strongylocentrotus_sp*	4 Benthos	Benthic	13	Peripheral
105	*Terebellides_stroemi*	4 Benthos	Benthic	10	Peripheral
106	*Thyasira_gouldi*	4 Benthos	Benthic	6	Peripheral
107	*Yoldiella_solidula*	4 Benthos	Benthic	8	Peripheral
108	*Agonus_cataphractus*	5 Fish	Benthic	11	Peripheral
109	*Amblyraja_radiata*	5 Fish	Benthic	49	Peripheral
110	*Ammodytes*_spp	5 Fish	Benthopelagic	38	Module connector
111	*Anarhichas_denticulatus*	5 Fish	Benthic	22	Peripheral
112	*Anarhichas_lupus*	5 Fish	Benthic	33	Module connector
113	*Anarhichas_minor*	5 Fish	Benthic	27	Peripheral
114	*Anisarchus_medius*	5 Fish	Benthic	4	Peripheral
115	*Arctozenus_risso*	5 Fish	Pelagic	7	Peripheral
116	*Argentina*_sp	5 Fish	Pelagic	11	Peripheral
117	*Artediellus_atlanticus*	5 Fish	Benthic	10	Peripheral
118	*Bathyraja_spinicauda*	5 Fish	Benthic	16	Peripheral
119	*Benthosema_glaciale*	5 Fish	Pelagic	6	Peripheral
120	*Brosme_brosme*	5 Fish	Benthic	13	Peripheral
121	*Careproctus*_sp	5 Fish	Benthopelagic	8	Peripheral
122	*Clupea_harengus*	5 Fish	Pelagic	58	Module connector
123	*Cottunculus_microps*	5 Fish	Benthic	6	Peripheral
124	*Cyclopterus_lumpus*	5 Fish	Benthopelagic	5	Peripheral
125	*Enchelyopus_cimbrius*	5 Fish	Benthic	4	Peripheral
126	*Etimopterus_spinax*	5 Fish	Benthic	5	Peripheral
127	*Gadiculus_argenteus*	5 Fish	Pelagic	14	Peripheral
128	*Gadus_morhua*	5 Fish	Benthopelagic	112	Network hub
129	*Gaidropsarus_argentatus*	5 Fish	Benthic	9	Peripheral
130	*Gasterosteus_aculeatus*	5 Fish	Pelagic	6	Peripheral
131	*Glyptocephalus_cynoglossus*	5 Fish	Benthic	8	Peripheral
132	*Hippoglossus_hippoglossus*	5 Fish	Benthic	22	Peripheral
133	*Hippoglossoides_platessoides*	5 Fish	Benthic	28	Peripheral
134	*Leptoclinus_maculatus*	5 Fish	Benthic	4	Peripheral

135	*Limanda_limanda*	5 Fish	Benthic	18	Peripheral
136	*Liparis_montagui*	5 Fish	Benthic	10	Module connector
137	*Lumpenus_lampretaeformis*	5 Fish	Benthic	17	Module connector
138	*Lycodes_esmarkii*	5 Fish	Benthic	9	Peripheral
139	*Lycodes_ gracicilis*	5 Fish	Benthic	4	Peripheral
140	*Lycodes_pallidus*	5 Fish	Benthic	15	Peripheral
141	*Macrourus_berglax*	5 Fish	Benthic	20	Peripheral
142	*Mallotus_villosus*	5 Fish	Pelagic	46	Peripheral
143	*Maurolicus_muelleri*	5 Fish	Pelagic	12	Peripheral
144	*Melanogrammus_aeglefinus*	5 Fish	Benthopelagic	88	Network hub
145	*Merlangius_merlangus*	5 Fish	Benthopelagic	34	Module connector
146	*Micromesistius_poutassou*	5 Fish	Pelagic	55	Module connector
147	*Microstomus_kitt*	5 Fish	Benthic	4	Module connector
148	*Molva_molva*	5 Fish	Benthic	11	Peripheral
149	Fish_larvae	5 Fish	Benthopelagic	30	Module connector
150	*Pleuronectes_platessa*	5 Fish	Benthic	34	Peripheral
151	*Pollachius_pollachius*	5 Fish	Pelagic	18	Peripheral
152	*Pollachius_virens*	5 Fish	Pelagic	47	Peripheral
153	*Rajella_fyllae*	5 Fish	Benthic	8	Peripheral
154	*Reinhardtius_hippoglossoides*	5 Fish	Benthopelagic	36	Peripheral
155	*Scomber_scombrus*	5 Fish	Pelagic	25	Peripheral
156	*Sebastes_norvegicus*	5 Fish	Benthopelagic	23	Peripheral
157	*Sebastes_mentella*	5 Fish	Benthopelagic	62	Module connector
158	*Sebastes*_spp	5 Fish	Benthopelagic	29	Peripheral
159	*Sebastes_viviparus*	5 Fish	Benthic	8	Module connector
160	*Somniosus_microcephalus*	5 Fish	Benthopelagic	39	Peripheral
161	*Squalus_acanthias*	5 Fish	Benthopelagic	14	Peripheral
162	*Triglops_murrayi*	5 Fish	Benthic	10	Peripheral
163	*Trisopterus_esmarkii*	5 Fish	Pelagic	35	Module connector
164	*Fratercula_arctica*	6 Birds	Pelagic	10	Peripheral
165	*Fulmarus_glacialis*	6 Birds	Pelagic	27	Peripheral
166	*Larus_argentatus*	6 Birds	Pelagic	5	Peripheral
167	*Larus_hyperboreus*	6 Birds	Pelagic	6	Peripheral
168	*Larus_marinus*	6 Birds	Pelagic	5	Peripheral
169	*Rissa_tridactyla*	6 Birds	Pelagic	10	Peripheral
170	*Uria_aalge*	6 Birds	Pelagic	7	Peripheral
171	*Uria_lomvia*	6 Birds	Pelagic	3	Peripheral
172	*Balaenoptera_acutorostrata*	7 Mammals	Pelagic	21	Peripheral
173	*Balaenoptera_physalus*	7 Mammals	Pelagic	22	Peripheral
174	*Erignathus_barbatus*	7 Mammals	Benthic	24	Peripheral
175	*Lagenorhynchus_albirostris*	7 Mammals	Pelagic	8	Peripheral
176	*Megaptera_novaeangliae*	7 Mammals	Pelagic	22	Peripheral
177	*Orcinus_orca*	7 Mammals	Pelagic	9	Peripheral
178	*Pagophilus_groenlandicus*	7 Mammals	Benthopelagic	17	Peripheral
179	*Phocoena_phocoena*	7 Mammals	Pelagic	32	Peripheral
180	*Physeter_macrocephalus*	7 Mammals	Pelagic	2	Peripheral

Table S2. List of taxa (in alphabetical order within each functional group) included in the Arctic region of the Barents Sea, and their functional affiliation, habitat use, degree (number of trophic interactions) and topological role.

Index	Taxon	Functional group	Habitat	Degree	Topological role
1	Detritus	1 Detritus	Benthopelagic	54	Module hub
2	Autothroph_flagellat	2 Basal taxa	Pelagic	15	Module connector
3	Bacteria_indet	2 Basal taxa	Benthopelagic	14	Peripheral
4	Diatom	2 Basal taxa	Benthopelagic	23	Peripheral
5	Heterotroph_flagellat	2 Basal taxa	Pelagic	13	Module connector
6	Ice_algae	2 Basal taxa	Pelagic	4	Peripheral
7	Macroalgae	2 Basal taxa	Benthic	3	Peripheral
8	Mixotroph_flagellates	2 Basal taxa	Pelagic	3	Peripheral
9	Phytoplankton_indet	2 Basal taxa	Pelagic	44	Network hub
10	Protozooplankton	2 Basal taxa	Pelagic	13	Peripheral
11	*Aglantha_digitale*	3 Zooplankton	Pelagic	2	Peripheral
12	*Apherusa_glacialis*	3 Zooplankton	Pelagic	8	Module connector
13	*Bolinopsis_infundibulum*	3 Zooplankton	Pelagic	7	Peripheral
14	*Calanus_finmarchicus*	3 Zooplankton	Pelagic	37	Module connector
15	*Calanus_glacialis*	3 Zooplankton	Pelagic	32	Module connector
16	*Calanus_hyperboreus*	3 Zooplankton	Pelagic	37	Module connector
17	*Clione_limacina*	3 Zooplankton	Pelagic	6	Peripheral
18	*Cyanea_capillata*	3 Zooplankton	Pelagic	16	Peripheral
19	*Dimophyes_arctica*	3 Zooplankton	Pelagic	2	Peripheral
20	*Eukrohnia_hamata*	3 Zooplankton	Pelagic	8	Peripheral
21	*Euphysa_flammea*	3 Zooplankton	Pelagic	6	Peripheral
22	*Fritillaria_borealis*	3 Zooplankton	Pelagic	4	Peripheral
23	*Gammarus_wilkitzkii*	3 Zooplankton	Pelagic	21	Module connector
24	*Limacina_helicina*	3 Zooplankton	Pelagic	15	Peripheral
25	*Mertensia_ovum*	3 Zooplankton	Pelagic	6	Peripheral
26	*Metridia_longa*	3 Zooplankton	Pelagic	21	Peripheral
27	*Metridia_lucens*	3 Zooplankton	Pelagic	4	Peripheral
28	*Microcalanus*_spp	3 Zooplankton	Pelagic	12	Peripheral
29	*Oikopleura*_spp	3 Zooplankton	Pelagic	7	Module connector
30	*Oikopleura_vanhoeffeni*	3 Zooplankton	Pelagic	5	Module connector
31	*Oithona_similis*	3 Zooplankton	Pelagic	7	Peripheral
32	*Onisimus_glacialis*	3 Zooplankton	Pelagic	9	Module connector
33	*Onisimus_nanseni*	3 Zooplankton	Pelagic	8	Module connector
34	*Pareuchaeta_glacialis*	3 Zooplankton	Pelagic	6	Peripheral
35	*Pareuchaeta*_spp	3 Zooplankton	Pelagic	10	Peripheral
36	*Pseudocalanus*_spp	3 Zooplankton	Pelagic	21	Peripheral
37	*Sagitta*_spp	3 Zooplankton	Pelagic	21	Peripheral
38	*Sarsia*_spp	3 Zooplankton	Pelagic	4	Peripheral
39	*Themisto_abyssorum*	3 Zooplankton	Pelagic	29	Peripheral
40	*Themisto_libellula*	3 Zooplankton	Pelagic	36	Peripheral
41	*Thysanoessa_inermis*	3 Zooplankton	Pelagic	33	Peripheral
42	*Thysanoessa_longicaudata*	3 Zooplankton	Pelagic	24	Peripheral

43	Actiniaria_g_sp	4 Benthos	Benthic	7	Peripheral
44	*Aglaophamus_malmgreni*	4 Benthos	Benthic	3	Peripheral
45	*Aphelochaeta_marioni*	4 Benthos	Benthic	4	Peripheral
46	*Arrhis_phyllonyx*	4 Benthos	Benthic	3	Peripheral
47	Ascidiacea_g_sp	4 Benthos	Benthic	6	Module connector
48	*Astarte*_sp	4 Benthos	Benthic	7	Peripheral
49	*Balanus*_sp	4 Benthos	Benthic	5	Peripheral
50	*Bathyarca_glacialis*	4 Benthos	Benthic	1	Peripheral
51	Bryozoa_indet	4 Benthos	Benthic	5	Peripheral
52	Benthos_larvae	4 Benthos	Benthopelagic	8	Module connector
53	*Buccinum*_sp	4 Benthos	Benthic	4	Peripheral
54	*Chaetozone*_sp	4 Benthos	Benthic	2	Peripheral
55	*Chionoecetes_opilio*	4 Benthos	Benthic	29	Peripheral
56	*Chlamys_islandica*	4 Benthos	Benthic	5	Peripheral
57	*Chone*_sp	4 Benthos	Benthic	4	Peripheral
58	*Ciona_intestinalis*	4 Benthos	Benthic	3	Peripheral
59	*Colus*_sp	4 Benthos	Benthic	2	Peripheral
60	*Crossaster_papposus*	4 Benthos	Benthic	5	Peripheral
61	*Ctenodiscus_crispatus*	4 Benthos	Benthic	3	Peripheral
62	*Electra_arctica*	4 Benthos	Benthic	4	Peripheral
63	Foraminifera	4 Benthos	Benthic	14	Module connector
64	*Galathowenia*_sp	4 Benthos	Benthic	3	Peripheral
65	*Gammaridae*_indet	4 Benthos	Benthic	19	Module connector
66	*Gonatus_fabricii*	4 Benthos	Pelagic	24	Peripheral
67	*Gorgonocephalus*_sp	4 Benthos	Benthic	2	Peripheral
68	*Heliometra_glacialis*	4 Benthos	Benthic	2	Peripheral
69	*Heteromastus_filiformis*	4 Benthos	Benthic	1	Peripheral
70	*Hyas*_sp	4 Benthos	Benthic	6	Peripheral
71	*Hydrozoa*_indet	4 Benthos	Benthic	2	Peripheral
72	*Maldane_sarsi*	4 Benthos	Benthic	3	Peripheral
73	*Mendicula_ferruginosa*	4 Benthos	Benthic	1	Peripheral
74	*Molpadia_borealis*	4 Benthos	Benthic	8	Peripheral
75	*Myriochele_herri*	4 Benthos	Benthic	2	Peripheral
76	*Nephtheidae*_sp	4 Benthos	Benthic	1	Peripheral
77	*Ophiacantha bidentata*	4 Benthos	Benthic	8	Peripheral
78	*Ophiocten sericeum*	4 Benthos	Benthic	4	Peripheral
79	*Ophiopholis aculeata*	4 Benthos	Benthic	9	Peripheral
80	*Ophiopleura borealis*	4 Benthos	Benthic	4	Peripheral
81	*Ophioscolex glacialis*	4 Benthos	Benthic	2	Peripheral
82	*Ophiura*_sp	4 Benthos	Benthic	19	Peripheral
83	*Ostracoda*_indet	4 Benthos	Benthic	15	Module connector
84	*Pagurus*_sp	4 Benthos	Benthic	7	Peripheral
85	*Pandalus_borealis*	4 Benthos	Benthic	41	Module connector
86	*Paramphinome_jeffreysii*	4 Benthos	Benthic	2	Peripheral
87	*Phascolion_strombus*	4 Benthos	Benthic	3	Peripheral
88	Polychaeta	4 Benthos	Benthic	34	Module connector

89	*Polynoidae*_indet	4 Benthos	Benthic	17	Peripheral
90	*Pontaster_tenuispinus*	4 Benthos	Benthic	1	Peripheral
91	Porifera_g_sp	4 Benthos	Benthic	7	Peripheral
92	*Prionospio_cirrifera*	4 Benthos	Benthic	2	Peripheral
93	Pycnogonida_g_sp	4 Benthos	Benthic	7	Peripheral
94	*Rhachotropis_macropus*	4 Benthos	Benthic	5	Module connector
95	*Rossia*_sp	4 Benthos	Benthic	9	Module connector
96	*Sabinea*_sp	4 Benthos	Benthic	15	Module connector
97	*Scalibregma_inflatum*	4 Benthos	Benthic	5	Peripheral
98	*Sclerocrangon_ferox*	4 Benthos	Benthic	16	Peripheral
99	*Similipecten_greenlandicus*	4 Benthos	Benthic	4	Peripheral
100	*Spiochaetopterus_typicus*	4 Benthos	Benthic	7	Peripheral
101	*Spiophanes_kroeyeri*	4 Benthos	Benthic	2	Peripheral
102	*Spirorbidae*_indet	4 Benthos	Benthic	2	Peripheral
103	*Strongylocentrotus*_sp	4 Benthos	Benthic	7	Peripheral
104	*Terebellides_stroemi*	4 Benthos	Benthic	7	Module connector
105	*Thyasira_gouldi*	4 Benthos	Benthic	1	Peripheral
106	*Urasterias_linckii*	4 Benthos	Benthic	1	Peripheral
107	*Amblyraja_hyperborea*	5 Fish	Benthic	19	Peripheral
108	*Anarhichas_lupus*	5 Fish	Benthic	22	Module connector
109	*Anisarchus_medius*	5 Fish	Benthic	3	Peripheral
110	*Arctogadus_glacialis*	5 Fish	Pelagic	7	Peripheral
111	*Artediellus_atlanticus*	5 Fish	Benthic	6	Peripheral
112	*Boreogadus_saida*	5 Fish	Benthic	42	Module connector
113	*Careproctus*_sp	5 Fish	Benthopelagic	8	Peripheral
114	*Cottunculus_microps*	5 Fish	Benthic	6	Peripheral
115	*Eumicrotremus_spinosus*	5 Fish	Benthic	8	Peripheral
116	*Gaidropsarus_argentatus*	5 Fish	Benthic	7	Peripheral
117	*Gymnelus*_spp	5 Fish	Benthic	7	Peripheral
118	*Gymnocanthus_tricuspis*	5 Fish	Benthic	8	Peripheral
119	*Hippoglossoides_platessoides*	5 Fish	Benthic	24	Module connector
120	*Icelus*_spp	5 Fish	Benthic	8	Module connector
121	*Leptagonus_decagonus*	5 Fish	Benthic	11	Module connector
122	*Leptoclinus_maculatus*	5 Fish	Benthic	2	Peripheral
123	*Liparis_fabricii*	5 Fish	Benthopelagic	7	Peripheral
124	*Liparis_gibbus*	5 Fish	Benthic	8	Peripheral
125	*Lumpenus_fabricii*	5 Fish	Benthic	4	Peripheral
126	*Lumpenus_lampretaeformis*	5 Fish	Benthic	10	Module connector
127	*Lycenchelys_kolthoffi*	5 Fish	Benthic	2	Peripheral
128	*Lycodes_eudipleurostictus*	5 Fish	Benthic	7	Peripheral
129	*Lycodes_pallidus*	5 Fish	Benthic	17	Peripheral
130	*Lycodes_reticulatus*	5 Fish	Benthic	10	Peripheral
131	*Lycodes_rossi*	5 Fish	Benthic	4	Peripheral
132	*Lycodes_seminudus*	5 Fish	Benthic	9	Peripheral
133	*Mallotus_villosus*	5 Fish	Pelagic	36	Module hub
134	*Myoxocephalus_scorpius*	5 Fish	Benthic	5	Peripheral

135	*Paraliparis_bathybius*	5 Fish	Benthic	2	Peripheral
136	Fish_larvae	5 Fish	Benthopelagic	21	Module connector
137	*Reinhardtius_hippoglossoides*	5 Fish	Benthopelagic	23	Peripheral
138	*Triglops_murrayi*	5 Fish	Benthic	7	Peripheral
139	*Triglops_nybelini*	5 Fish	Benthic	4	Module connector
140	*Triglops_pingelii*	5 Fish	Benthic	10	Module connector
141	*Ulcina_olrikii*	5 Fish	Benthic	2	Peripheral
142	*Alle_alle*	6 Birds	Pelagic	26	Peripheral
143	*Fulmarus_glacialis*	6 Birds	Pelagic	20	Module connector
144	*Larus_hyperboreus*	6 Birds	Pelagic	3	Peripheral
145	*Rissa_tridactyla*	6 Birds	Pelagic	6	Peripheral
146	*Uria_lomvia*	6 Birds	Pelagic	2	Peripheral
147	*Balaenoptera_acutorostrata*	7 Mammals	Pelagic	13	Peripheral
148	*Balaenoptera_physalus*	7 Mammals	Pelagic	13	Peripheral
149	*Cystophora_cristata*	7 Mammals	Pelagic	13	Peripheral
150	*Delphinapterus_leucas*	7 Mammals	Pelagic	9	Peripheral
151	*Erignathus_barbatus*	7 Mammals	Benthic	30	Module connector
152	*Lagenorhynchus_albirostris*	7 Mammals	Pelagic	3	Peripheral
153	*Megaptera_novaeangliae*	7 Mammals	Pelagic	11	Peripheral
154	*Monodon_monoceros*	7 Mammals	Pelagic	7	Peripheral
155	*Odobenus_rosmarus*	7 Mammals	Benthic	15	Peripheral
156	*Orcinus_orca*	7 Mammals	Pelagic	6	Peripheral
157	*Pagophilus_groenlandicus*	7 Mammals	Benthopelagic	16	Peripheral
158	*Phoca_hispida*	7 Mammals	Benthopelagic	22	Module connector
159	*Ursus_maritimus*	7 Mammals	Benthopelagic	5	Peripheral

Table S3. Abbreviations, names and short definitions of food web metrics calculated in this paper.

Abbreviation	Full name	Definition	Reference
S	Species richness	Number of taxa (nodes) in a food web	Dunne (2009)
L	Trophic links	Number of trophic (feeding) interactions (links)	Dunne (2009)
LD	Link density	Mean number of links per species	Dunne (2009)
C	Connectance	Proportion of all possible links realized ($C=L/S^2$)	Dunne (2009)
% - Omni	Omnivores	Fraction of taxa that feed on resources on more than one trophic level	Petchey (2008)
% - Can	Cannibals	Fraction of taxa that feed on themselves	
% in loops	Species in loops	Fraction of taxa that occur in loops. A loop describes a pathway of interactions from a certain species without visiting the species more than once	Williams (2010)
MeanPath	Mean shortest path length	Mean shortest path of feeding links connecting each pair of taxa in a food web	Dunne (2009)
MeanOmni	Mean level of omnivory	Level of omnivory of each species is the standard deviation of the SWTL of its resources.	Petchey (2008)
MeanSWTL	Mean short-weighted Trophic level	Mean of all short weighted paths + 1 from base to each species of interest	Williams & Martinez (2004)
MeanClust	Mean clustering	The probability that two neighbours of a species are neighbours themselves	Girvan & Newman (2002)
Mod	Modularity	Modularity refers to subgroups of species interacting more with each other than with species from other subgroups	Newman & Girvan (2004)

References: Dunne, J. A. 2009 Food webs. In Complex Networks and Graph Theory section of the Encyclopedia of Complexity and Systems Science, pp. 3661–3682. Ed. by R. A. Meyers. Springer, New York.; Petchey, O. L., Beckerman, A.P., Riede, J.O, Warren, P.H. 2008 Size, foraging and food web structure. PNAS. **100**, 614-622; Williams, R. 2010 Network 3D software. *Microsoft Research, Cambridge, UK*; Williams R. J., Martinez, N. D. 2004 Limits to trophic levels and omnivory in complex food webs: theory and data. *Am Nat*. 163, 458-468; Girvan, M., Newman, M. E. J. 2002 Community structure in social and biological networks *Proc. Natl. Acad. Sci.* **90**, 7821-7826; Newman, M. E., Girvan, M. 2004 Finding and evaluating community structure in networks. *Phys. Rev. E.* **69**, 026113.

Table S4. List of 51 fish taxa (in alphabetical order) used to calculate the degree centrality maps. For some fish the occurrence data at station level had a lower taxonomic resolution (e.g *Zoarcidae* family rather than genus and species level) than in the food web matrix meta-web. For these taxa, we calculated the mean degree centrality of the taxonomic level of interest (e.g. family) based on the degree centrality of the member species.

Amblyraja hyperborea, Amblyraja radiata, Anarhichas denticulatus, Anarhichas lupus, Anarhichas minor, Anisarchus medius, Argentina sp, *Artediellus atlanticus, Bathyraja spinicauda, Brosme brosme, Careproctus* sp., *Cottunculus microps, Cyclopterus lumpus, Enchelyopus cimbrius, Eumicrotremus spinosus, Gadiculus argenteus, Gadus morhua, Gaidropsarus argentatus, Glyptocephalus cynoglossus, Gymnocanthus tricuspis, Hippoglossus hippoglossus, Hippoglossoides platessoides, Icelus* spp., *Leptagonus decagonus, Leptoclinus maculatus, Limanda limanda, Liparis montagui, Lumpenus fabricii, Lumpenus lampretaeformis, Lycodes esmarkii, Lycodes gracilis, Macrourus berglax, Melanogrammus aeglefinus, Merlangius merlangus, Microstomus kitt, Micromesistius poutassou, Molva molva, Myoxocephalus Scorpius, Pleuronectes platessa, Pollachius virens, Rajella fyllae, Reinhardtius hippoglossoides, Sebastes mentella, Sebastes* spp., *Sebastes viviparous, Triglops murrayi, Triglops nybelini, Triglops pingelii, Trisopterus esmarkii, Ulcina olrikii, Zoarcidae (include these taxa: Gymnelus* spp., *Lycodes eudipleurostictus, Lycodes pallidus, Lycodes reticulatus, Lycodes rossi, Lycodes seminudus)*
